# Draft Genome Sequence of Cellulolytic and Xylanolytic *Paenibacillus* sp. A59, Isolated from Decaying Forest Soil from Patagonia, Argentina

**DOI:** 10.1128/genomeA.01233-15

**Published:** 2015-10-22

**Authors:** Silvina Ghio, Alfredo I. Martinez Cáceres, Paola Talia, Daniel H. Grasso, Eleonora Campos

**Affiliations:** a Instituto de Suelos, CIRN, Instituto Nacional de Tecnología Agropecuaria (INTA), Buenos Aires, Argentina; b Instituto de Biotecnología, CICVyA, Instituto Nacional de Tecnología Agropecuaria (INTA), Buenos Aires, Argentina

## Abstract

*Paenibacillus* sp. A59 was isolated from decaying forest soil in Argentina and characterized as a xylanolytic strain. We report the draft genome sequence of this isolate, with an estimated genome size of 7 Mb which harbor 6,424 coding sequences. Genes coding for hydrolytic enzymes involved in lignocellulose deconstruction were predicted.

## GENOME ANNOUNCEMENT

Microbial cellulases have become important for many applications in food processing, animal feed, pulp and paper industries, and second generation biofuels production (1). In particular, bacteria are promising sources of enzymes for biomass bioconversion. Members of the *Paenibacillus* genus have been isolated from soil and plant related sources (2) and have been reported to produce many extracellular enzymes for industrial applications (3).

In our study, a novel cellulolytic and xylanolytic strain, named *Paenibacillus* sp. A59, was isolated from a previously characterized cellulolytic bacterial consortium, obtained from decaying forest soil in the Patagonia region, Argentina (4). These bacteria are Gram-positive endospore-forming, facultative anaerobe bacillus. Based on 16S rRNA gene sequence analysis, it formed a cluster with *P. taichungensis* (gi|343199050|) and *P. pabuli* (gi|343200166|). A crude enzymatic extract of *Paenibacillus* sp. A59 was capable of converting pre-treated sugarcane agricultural residue to simple sugars such as xylobiose, xylose, cellobiose, and arabinose, demonstrating its potential for lignocellulosic biomass deconstruction (S. Ghio et al., unpublished results).

Bacterial genomic DNA was obtained from a 24 h culture in Luria Bertoni broth, by a commercial extraction kit (Wizard Genomic DNA Extraction kit, Promega). Genome sequencing was performed using Illumina MiSeq platform. A total of 4,201,470 paired-end reads were generated, with an average length of 250 bp, achieving 110-fold coverage of the genome. The raw reads were trimmed using Trimmomatic version 0.33 (5) and assembled *de novo* using Velvet version 1.2.10 (6), producing 83 contigs with an accumulated length of 7,087,589 (*N*_50_ 317,284 bp) and an average G+C content of 46%.

The functional annotation was performed with the Rapid Annotations using Subsystems Technology (RAST) server, version 2.0 (7) and NCBI Prokaryotic Genome Annotation Pipeline (http://www.ncbi.nlm.nih.gov/genome/annotation_prok/), resulting in 6,424 predicted coding sequences, 39% of which were assigned to 447 sub-systems. Also, 48 RNA sequences were identified: 46 tRNAs and one copy of 23S/5S and 16S rRNA genes. A comparison of *Paenibacillus* sp. A59 genome sequence with others available in the RAST server resulted in *Geobacillus* sp. Y412MC10 (score 524) and *Paenibacillus* sp. oral taxon 786 strain D14 (score 461) as closest neighbors. Several genes coding for potential glycoside hydrolases (GHs), as classified by the CAZY database (8), were found. Among them, 4 endoxylanases (GH10:3, GH11:1), 2 endoglucanases (GH5:1, GH9:1), 2 cellobiohydrolases (GH6:1, GH48:1), 13 β-xylosidases/arabinofuranosidases (GH43:12, GH52:1), and 7 β-glucosidases (GH1:1, GH3:6) were the most relevant enzymes identified for lignocellulosic biomass deconstruction. These preliminary results correlate with the observed enzymatic activity of *Paenibacillus* sp. A59 and highlight the role of *Paenibacillus* genus as a source of (hemi) cellulolytic enzymes for biotechnological and industrial applications.

### Nucleotide sequence accession numbers.

The draft genome sequence of *Paenibacillus* sp. A59 has been deposited in DDBJ/EMBL/GenBank under the accession no. LITU00000000. The version described in this paper is LITU01000000.
